# The German version of the Nottingham Clavicle Score is a reliable and valid patient-reported outcome measure to evaluate patients with clavicle and acromioclavicular pathologies

**DOI:** 10.1007/s00167-022-07129-6

**Published:** 2022-08-29

**Authors:** Sebastian Scheidt, Jakob Zapatka, Richard Julius Freytag, Malin Sarah Pohlentz, Matteo Paci, Koroush Kabir, Christof Burger, Davide Cucchi

**Affiliations:** 1 Department of Orthopaedics and Trauma Surgery, University Hospital Bonn, Venusberg-Campus 1, 53127 Bonn, Germany; 2 Department of Internal Medicine, Helios Klinikum Bonn/Rhein-Sieg, Von-Hompesch-Str. 1, 53123 Bonn, Germany; 3 Unit of Functional Rehabilitation, Azienda USL Toscana Centro, Via di San Salvi, 12, Firenze, Italy; 4 Centre of Trauma Surgery, Orthopaedics and Sport Medicine, Helios University Hospital Wuppertal, Heusnerstraße 40, 42283 Wuppertal, Germany

**Keywords:** Clavicle, Acromioclavicular joint, Nottingham Clavicle Score, Translation, Validation, Adaptation, Cross-cultural, Patient-reported, German

## Abstract

**Purpose:**

The Nottingham Clavicle Score (NCS) is a patient-reported outcome measure developed to evaluate treatment results of clavicle, acromioclavicular and sternoclavicular joint pathologies. Valid, reliable and user-friendly translations of outcome measure instruments are needed to allow comparisons of international results. The aim of this cross-sectional study was to translate and adapt the NCS into German and evaluate the psychometric properties of the German version.

**Methods:**

The translation and cross-cultural adaptation of the NCS were completed using a ‘translation–back translation” method and the final version was administered to 105 German-speaking patients. The psychometric properties of this version (NCS-G) were evaluated in terms of feasibility, reliability, validity and sensitivity to change.

**Results:**

No major differences occurred between the NCS translations into German and back into English, and no content- or linguistic-related difficulties were reported. The Cronbach’s alpha for the NCS-G was 0.885, showing optimal internal consistency. The Intraclass Correlation Coefficient for test–retest reliability was 0.907 (95% CI 0.844–0.945), with a standard error of measurement of 5.59 points and a minimal detectable change of 15.50 points. The NCS-G showed moderate to strong correlation with all other investigated scales (Spearman correlation coefficient: qDASH: *ρ* =  – 0.751; OSS: *ρ* = 0.728; Imatani Score: *ρ* = 0.646; CMS: *ρ* = 0.621; VAS: *ρ* =  – 0.709). Good sensitivity to change was confirmed by an effect size of 1.17 (95% CI 0.89–1.47) and a standardized response mean of 1.23 (95% CI 0.98–1.45).

**Conclusions:**

This study demonstrated that NCS-G is reliable, valid, reproducible and well accepted by patients, showing analogous psychometric properties to the original English version.

**Level of evidence:**

Level III.

**Supplementary Information:**

The online version contains supplementary material available at 10.1007/s00167-022-07129-6.

## Introduction

Clavicle fractures and acromioclavicular joint (ACJ) dislocations are frequent injuries affecting mostly young, active patients [18, 25, 33–35, 39]. Together with the rarer injuries of the sternoclavicular joint (SCJ) and with degenerative conditions of both ACJ and SCJ affecting elderly patients, these pathologies may impair everyday- and professional- and recreational-life as well as the function of the whole upper limb, chest external appearance, and cosmesis [15, 19, 24, 27, 28, 38, 42]. The Nottingham Clavicle Score (NCS) is a patient-reported outcome measure (PROM), specifically designed to assess clinical outcome after injuries and degenerative pathologies of the clavicle, ACJ and SCJ [9]. This and other PROMs have been developed during the last decades to enable patients to self-assess information about their functional status, symptom and perceived well-being [2, 8, 16, 23]. These measurement tools also add another dimension to clinical outcome evaluation, traditionally focused on objective parameters such as functional or radiographic analyses [32, 36].

Outcomes of different pathologies and interventions should be assessed through an appropriate combination of different PROMs. In particular, recent international recommendations advise combining an anatomical-district score and a disease-specific score to thoroughly assess a pathologic condition with PROMs. To fulfill this task, the chosen PROMs should be validated, have large diffusion in the international scientific world and be made available in many languages through an appropriate and rigorous process of translation and cross-cultural adaptation [3, 36].

The NCS was developed as a disease-specific score and can thus well complete a core set of outcome measures for patients with injuries and degenerative pathologies of the clavicle, ACJ and SCJ. Currently, besides the original English version, a single validated translation is available into the Italian language [47].

The aim of this cross-sectional study was to translate and cross-culturally adapt the NCS into German and to evaluate the psychometric properties of the German version in terms of feasibility, reliability, validity and sensitivity to change, to give German-speaking clinicians and patients a subjective method to evaluate shoulder impairment in context of lesions of the clavicle, ACJ and SCJ.

The hypothesis of the study was that the German version of the NCS would display similar psychometric properties to those of the original English version, in particular in terms of reliability (Cronbach’s alpha as measure of internal consistency).

## Materials and methods

### Authorizations and ethic committee approval

The developers of the original NCS provided authorization to use the original version of the NCS prior to study begin. The study was conducted according to the principles of the declaration of Helsinki and was approved by the local ethic committee (Ethics Committee of the Medical Faculty, University Hospital Bonn, University of Bonn, Building 74/4th floor, Venusberg-Campus 1, 53,105 Bonn, Germany, No. ID 419/19). Written informed consent was received from all subjects before participation.

### The Nottingham Clavicle Score

The NCS is a 10-item PROM, specifically designed to measure outcomes after injuries and degenerative pathologies of the clavicle, ACJ and SCJ. The explored dimensions are pain (four items), strength and functional abilities (two items), cosmetic satisfaction (one item), mechanical symptoms such as movements or clicking (one item) and neurological symptoms in the upper limb such as tingling, numbness, heaviness and dragging sensations (two items). The final score ranges from 20 to 100 points and can be graded as excellent (80–100), good (60–79), fair (40–59) or poor (< 40) [9, 47].

### Translation and cross-cultural adaptation process

The German translation and cultural adaptation of the scale were completed according to the stages recommended by Beaton et al. using a “translation–back translation” method [5], consisting of the following steps:

1. After authorization by the original developers of the NCS, the original English version of the scale was translated into the German language by two, independent, bilingual translators, fully competent in both languages, one of which with specialized competence on medical procedures.
2. The two translations were evaluated by an expert panel of three orthopedic surgeons, a physical therapist with special interest in shoulder rehabilitation and two researchers, active in the field of orthopedics, sports medicine and rehabilitation. Both versions were merged to obtain a best fitting translation, after consensual resolution of all points of disagreement.
3. A third professional translator, blinded to the original document and to the validation process and not involved in the creation of the first version of the translation, performed a back translation, which was then evaluated to reveal inconsistencies with the original English version.
4. The definitive translation was evaluated by the aforementioned expert committee to validate for content, semantic, technical, criterion, and conceptual equivalence. The obtained document (NCS-G, pre-final version) was considered equivalent to the original version.
5. To assess content validity, confirm the comprehensibility and to search for unanswered items and possible problems of interpretation, the pre-final version of the scale underwent a pilot testing with 30 native German-speaking subjects enrolled as healthy volunteers, with no previous history of clavicle or shoulder trauma or diseases. To ensure that the questions would not be considered as too conceptual and that non-health-care-professionals would understand the questions, the healthy volunteers were encouraged to leave a comment, in case of difficult understanding. The time necessary to complete the questionnaire and any difficulty encountered in answering the questions were recorded.
6. After the pilot test, minor modifications were made to the unified translation, according to consensus among the expert panel, and the final version (NCS-G) was then approved by the authors (Appendix 1).
7. The original developer of the NCS were finally notified about the completion of the translation and cross-cultural adaptation process and approved the final German version.

### Patients and outcome measures

A monocenter, cross-sectional study was designed to assess the feasibility, reliability, validity and responsiveness of the final version of the NCS-G according to the COSMIN checklist [31].

Between January 2020 and January 2022, German-speaking patients older than 16 years referring to the investigation center for diagnosis and treatment of injuries and degenerative pathologies of the clavicle, ACJ and SCJ without associated shoulder injuries or pathologies were prospectively screened for enrollment.

A total of 105 patients were included. Demographic data of the included patients are reported in Table 1. The spectrum of patients’ pathologies and the performed surgical treatments (76.2% of the cases) are summarized in Figures s1 and s2 (Supplementary materials).

**Table 1:** Patient’s demographics

Group	Overall	Test– retest reliability cohort	Sensitivity to changes cohort
No. of patients	105	53	50
Age at follow-up (years)	40.00 ± 17.58 36.90 [24.74 – 52.52]	44.90 ± 20.52 37.86 [28.00 – 62.11]	41.67 ± 16.90 39.25 [28.08 – 53.04]
BMI (kg/m^2^)	26.62 ± 11.84 24.52 [22.13 ± 27.65]	24.86 ± 4.32 24.31 [21.97 ± 27.55]	25.46 ± 5.82 24.22 [22.25 ± 27.20]
Gender (F/M)	0.29/0.71	0.40/0.60	0.22/0.78
Treated side (L/R)	0.59/0.41	0.53/0.47	0.60/0.40
Dominant side (L/R)	0.12/0.88	0.25/0.75	0.18/0.82

Data are reported as mean ± SD and median [Q1-Q3] or number of cases/patients (percentage/frequency). *BMI* body mass index, *No.* number, *Q1* first quartile, *Q3* third quartile, *SD* standard deviation, *F* females, *M* males, *L* left, *R* right

All patients underwent a standardized clinical outcome evaluation: a combination of a quality of life assessment instrument (EQ-5D-5L) with a district-specific PROM, a shoulder- and a clavicle-specific PROMs and a pain assessment tool (Visual Analogue Scale, VAS [41]), were used. Following local recommendations, the Disabilities of the Arm, Shoulder and Hand questionnaire (DASH) was collected as anatomical-district score, in its concept-retention form (*QuickDASH*, qDASH) [6, 20, 36]. The NCS-G was used as clavicle-specific PROM and the Oxford Shoulder Score (OSS) as shoulder-specific PROM. During the clinical evaluation, the Constant-Murley Score (CMS) and the Imatani scores, used in the validation of the original version of the NCS, were collected by an orthopaedic surgeon and isometric strength in shoulder abduction was measured [9, 12, 21]. All measures were performed in triplicate with a dynamometer (IsoForceControl ® EVO2, Medical Device Solutions AG, Oberburg, Switzerland). Appendix 2 provides extensive description of the scores used during this clinical trial.

### Assessment of the psychometric properties of the NCS-G

*Feasibility:* feasibility was evaluated by counting the number of missing responses and dividing this by the total number of collected items.

*Reliability:* the internal consistency of the scale was evaluated by calculation of Cronbach’s alpha based on the correlation among the 10 items [46]. To explore the test–retest reliability, the NCS-G was re-administrated to at least 50% of the planned patients 14 ± 7 days after the first administration. This interval was considered short enough to assume an unchanged clinical condition and long enough to forget prior answers.

*Validity:* similarly to the development process of the original version, the NCS-G was compared with the EQ-5D-5L index value and its subscales, the Imatani score, the OSS and the CMS to estimate construct validity [12, 13, 21]; additionally, comparison with the qDASH was performed [20].

*Floor and ceiling effects:* floor and ceiling effects were assessed by calculating the number of patients who obtained the best or worst possible scores. If more than 15% of subjects achieved the lowest or highest possible score, floor or ceiling effects were considered to be present [29, 44].

*Sensitivity to change:* to evaluate the ability of the NCS-G to detect clinically relevant change over a period of time, at least 50% of the planned patients were recalled to receive a complete re-evaluation no less than 7 weeks after treatment begin [11, 26].

### Statistical analysis

The test–retest reliability of the NCS-G was estimated by calculation of the Intraclass Correlation Coefficient (ICC_2,1_) with 95% confidence intervals (CI). ICC values were interpreted according to the guidelines of Fitzpatrick et al. [17], i.e., ICC = 0.70 and ICC = 0.90 being considered as minimum acceptable levels for measures to be used when assessing groups or individuals, respectively. Bland–Altman plots were used to depict the congruence of scores.

Based on ICC vales, the standard error of measurement (SEM) and the minimal detectable change (MDC) were calculated. The scale Cronbach’s alpha coefficient was calculated to measure internal consistency and values from 0.70 to 0.95 were considered as acceptable [7].

Construct validity was estimated by calculation of the Spearman rank correlation coefficients for the NCS-G total score and the qDASH, the OSS, the Imatani score, the CMS, the VAS, the EQ-5D-5L index value and its subscales as well as the range of motion in abduction, flexion, external and internal rotation.

Cohen effect size (ES) and standardized response mean (SRM), were computed as standardized indicators of power of an instrument to detect true change (sensitivity to change), with larger values indicating higher sensitivity to change (< 0.20: trivial; ≥ 0.20 to < 0.50: small; ≥ 50 to < 0.80: moderate and ≥ 0.80: large) [11, 26].

Data analyses (M.P.) were performed using the SPSS statistical package 20.0 for Windows.

The sample size was defined as *n* = (10⋅*i*), where *i* represents the number of items of the investigated PROM [46], and the number of patients used to evaluate intra-rater reliability was calculated with the equation *n* = (5⋅*i*) [37, 46]. Following these equations, a sample size of 100 and a retest size of 50 were required for this study.

## Results

### Translation, cross-cultural adaptation, pre-test phase

No content- or linguistic-related difficulties were documented for the process of translation from the original version into German and back to English. The final version was considered free of cross-cultural inconsistencies, so that the authors considered all questions applicable to a German-speaking population. None of the 30 volunteers (age: 35.5 ± 13.8; high-school degree: 46.7%; university degree: 30%; apprenticeship/professional school: 23.3%) reported any difficulties processing the questionnaires, due to language problems or redundancy. The mean time needed to complete the questionnaire was 3.5 ± 1.9 min. The NCS-G is shown in Appendix 1.

### Psychometric properties of the NCS-G

*Feasibility:* 20 missing responses were observed over a total of 1050 (1.9%). Item 7 (“Have you been happy about the appearance of your collarbone area?”) was the one reporting the highest frequency of missing responses (*n* = 6).

**Fig. 1:**
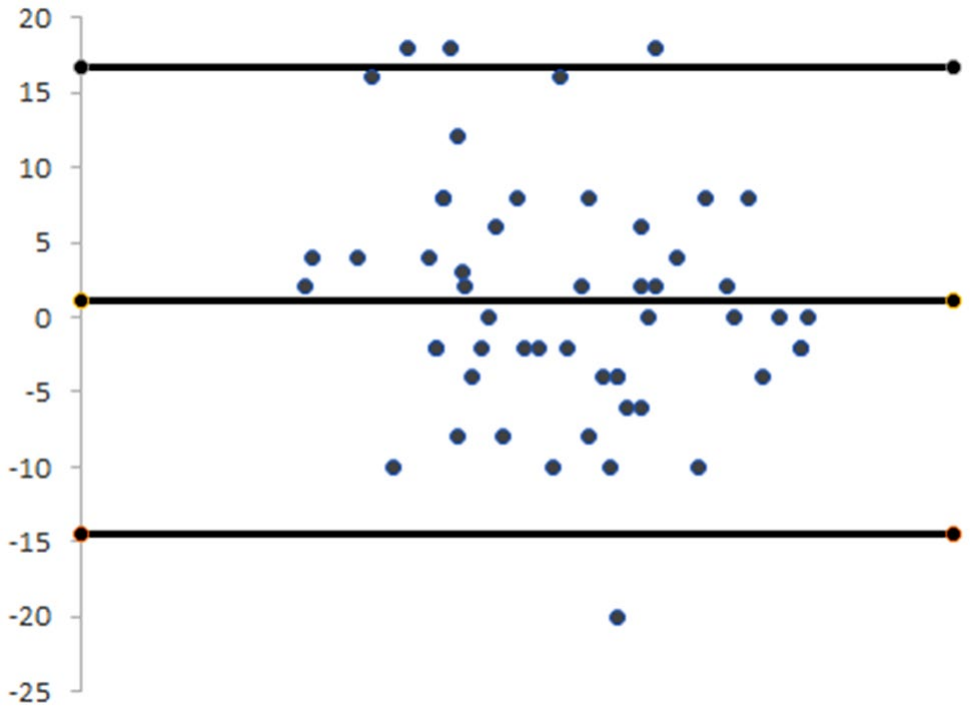
Bland–Altman plot depicting the congruence of the NCS-G. Incongruencies are indicated by values outside the 95% confidence intervals (solid upper and lower lines). Absence of systematic errors is confirmed by the position of the solid mean difference line (solid central line) and by the distribution of the values around the “0”

*Reliability:* the retest was completed by 53 patients at an average test–retest interval of 17.8 days, scoring 66.0 ± 19.2 points in the first attempt and 67.1 ± 17.6 points at the retest. For the test–retest reliability the ICC_2,1_ was 0.907 (95% CI 0.844–0.945). The SEM and the MCD were 5.59 and 15.50, respectively. The internal consistency of the scale showed a Cronbach’s alpha of 0.885. Figure 1 shows the Bland–Altman plot of the reliability data collected in this cohort.

*Construct validity:* the NCS-G showed moderate to strong correlation with all other investigated scales (Spearman correlation coefficient: qDASH: *ρ* =  – 0.751; OSS: *ρ* = 0.728; Imatani: *ρ* = 0,646; CMS: *ρ* = 0.621; VAS: *ρ* =  – 0.709), and this correlation was throughout statistically significant with *p* < 0.001. As expected, only moderate to low correlation was present between this clavicle-specific score and the range of motion of the shoulder joint in the different planes (0.446 < *ρ* < 0.472) and the NCS and the EQ-5D-5L index value (*ρ* = 0.406). Correlation with the EQ-5D-5L subscales varied between weak (mobility) and moderate (pain/discomfort) reflecting the characteristics of the original publication [9] (Table 2).

**Table 2:** Spearman analysis performed to evaluate correlation between the NCS-G and the subscales of the EQ-5D-5L

	Correlation coefficient	Significance (2-tailed)
Mobility	– 0.289	0.003
Self-care	– 0.467	< 0.001
Usual activities	– 0.576	< 0.001
Pain/discomfort	– 0.651	< 0.001
Anxiety/depression	– 0.315	0.001

*Floor and ceiling effects*: six patients (5.7%) reached full marks and no patient obtained the minimum possible score; neither floor nor ceiling effects could be observed.

*Sensitivity to change:* 50 patients were further re-evaluated after an average interval of 6 months to evaluate the ability of the NCS-G to detect clinically relevant changes, scoring 60.8 ± 16.9 points in the first and 78.9 ± 13.9 points in the second evaluation. The ES and SRM of all investigated outcome measures are reported in Table 3.

**Table 3:** Cohen effect size and standardized response mean of the NCS-G and the other investigated outcome measures

	ES using pooled SD (95% CI)		SRM (95% CI)	
NCS-G	1.17	(0.89–1.47)	1.23	(0.98–1.45)
qDASH	– 1.15	(– 1.50 to – 0.84)	– 1.07	(– 1.37 to – 0.82)
OSS	1.26	(0.92 to 1.65)	1.03	(0.81 to 1.27)
Imatani Score	1.35	(0.95 to 1.83)	1.04	(0.70 to 1.41)
VAS for pain	– 1.00	(– 1.35 to – 0.68)	– 0.85	(– 1.13 to – 0.55)
EQ-5D-5L index value	0.99	(0.70 to 1.29)	0.76	(0.58 to 0.96)

*CI* confidence interval, *ES* effect size, *NCS-G* German version of the Nottingham Clavicle Score, *OSS* Oxford Shoulder Score, *qDASH* Disabilities of the Arm, Shoulder, and Hand Questionnaire, concept-retention version, *SD* standard deviation, *SRM* standardized response mean, *VAS* Visual Analogue Scale

## Discussion

The main finding of this study is that the proposed German version of the NCS is reliable, valid, reproducible and well accepted by patients, showing analogous psychometric properties to the original English version.

The NCS has been designed and validated by the Nottingham Shoulder & Elbow Unit in 2013 and is to date the only internationally widespread disease-specific PROM available to collect outcomes after clavicle, ACJ and SCJ injuries, receiving the endorsement of national societies, due to its ability to encompass all previously mentioned aspects affecting patients’ life after clavicle, ACJ and SCJ pathologies [1, 43]. The NCS was developed to overcome some limitation of previous assessment tools, such as the OSS and the CMS, which are not as specific and give only small weighting for sports, recreation or cosmetic appearance. As other PROMS, the NCS does not require a clinician to be present, takes only few minutes to complete, is standardized and assesses function and well-being as perceived by the patient [9]. Currently, the original version of the NCS has been translated, adapted to the Italian language and validated by Vascellari et al. on a cohort of 63 Italian-speaking patients suffering injuries of the ACJ and the clavicle [47] and further validated with a cohort of 36 patients with clavicle shaft fractures treated with flexible titanium nails by Vishwanathan et al. [48].

For the first time, this study presents a translation, cross-cultural adaptation, and validation of the German version of the NCS, opening for the use of this score to Europe’s largest professional linguistic basin, accounting for clinicians and patients from Germany, Austria, Switzerland, Belgium, Luxemburg and Liechtenstein (approximately 100 million of German-speaking inhabitants). The translation respected strict international guidelines and the validation process was based on a broad patient basis and adhered to the structure of the original English version, allowing for comparison [5]. The previously published Italian version of the NCS respects also the same translation and validation structure, with the exception of the use of the SF-36 instead of the EQ-5D-5L as general quality of life questionnaire [47].

The translation and adaptation into German did not need major cultural adaption and we found the NCS-G to be valid, reliable, reproducible and well accepted by patients, showing analogous psychometric properties to the original English version and to the Italian translation.

All three studies revealed an optimal internal consistency (Cronbach α: NCS = 0.87; NCS-IT = 0.86; NCS-G = 0.885), supporting the strong homogeneity among the items on a test, without risk of redundancy. The ICC for test–retest reliability was similar between Italian and German versions (NCS-IT = 0.981; NCS-G: 0.907) supporting the use of the tool to assess individuals, according to the guidelines of Fitzpatrick et al., in both cases [17]. Construct validity also appeared also to be similar between the Italian and German translations, both showing moderate to strong correlation all other investigated anatomical-district and organ-specific upper limb scores (Spearman correlation coefficient: NCS-IT – qDASH: *ρ* = – 0.87; NCS-G – qDASH: *ρ* =  – 0.751; NCS-IT – OSS: *ρ* = 0.84; NCS-G – OSS: *ρ* = 0.728). As expected, a low correlation was found between NCS and EQ-5D-5L index values, since the first one is a disease-specific measure, while the other is a global and generic questionnaire. Similar finding was reported by Vascellari et al., correlating the NCS with the SF-36, another unspecific tool to assess quality of life [47].

As in the original publication, two clinician-reported outcome measures were also collected, the Imatani Score, and the CMS; similarly, we could also identify a less strong correlation of these scores with the NCS-G (Imatani: *ρ* = 0.646; CMS: *ρ* = 0.621) [9]. A recent retrospective study could identify a slightly stronger correlation of the NCS with the CMS in a selected subgroup of 58 patients undergoing ACJ stabilization procedures (*ρ* = 0.79) [14]. Neither floor nor ceiling effects could be observed in our validation and in that by Vishwanathan et al. [48]. The NCS-G was able to detect the change after the index visit, with high ES and SRM, slightly inferior to that reported in the original publication (ES = 1.92) and in the study by Vishwanathan et al. (ES and SRM of 1.8 and 2.6, respectively)[48].

Few other scores have been proposed, to assess clinical outcomes after injuries to the clavicle, ACJ, and SCJ or treatment of chronic pathologies of these structures; however, none of them reaching wide international diffusion. Jubel et al. designed and validated in Germany a multidimensional score to evaluate outcomes after clavicular midshaft fractures, containing subjective and objective element as well as radiographic assessment for fracture healing [22]. The diffusion of this score has remained limited to German-speaking countries due to the lack of an English translation and the need of a trained clinician to collect data. Some specific uni- or multidimensional scores for ACJ dislocations have been developed more than 30 years ago, before the development of strict criteria to create such outcome collection tools, as the Imatani and the Taft scores [21, 45]. More recently, a multidimensional clinician-reported outcome score called Acromioclavicular Joint Instability Score was described by Scheibel et al. [30, 40] and a multidimensional PROM called Specific AC Score by Barwood et al. [4, 10].

The strengths of this study are the presence of a power analysis guaranteeing sound statistical results and the adherence to international guidelines throughout the design and conduction process [5, 31]. Limitations of this study include the choice of a slightly different patient population from that enrolled in the original publication, in which ACJ osteoarthritis was the dominant pathology. Our choice reflects that of Vascellari et al., who validated the Italian version on a cohort of patients who had received surgical treatment for injuries of the ACJ. A possible source of bias when comparing this validation study to the previous ones regards the choice of different retest intervals to evaluate test–retest reliability and sensitivity to change. Furthermore, the use of the concept-retention version of the DASH score was preferred to the full version to reduce the patient’s burden in completing the set of PROMs. Since both the *Quick*DASH and the full DASH outcome measure are valid, reliable, responsive and can be used for clinical and research purposes, we opted for the first version [6]. Finally, some patients were excluded from the analysis of the CMS due to pain or functional limitations impeding abduction > 90°; this can bias the assessment of the construct validity when comparing the NCS-G to the CMS.

## Conclusions

The proposed German version of the NCS is reliable, valid, reproducible, sensible to changes and well accepted by patients, showing analogous psychometric properties to the original English version. These properties make it recommended for outcome assessment after injuries and degenerative pathologies of the clavicle, ACJ and SCJ in German-speaking countries.

## Supplementary Information

Below is the link to the electronic supplementary material.

Supplementary file1 (PDF 120 KB)

Supplementary file2 (DOCX 20 KB)

Supplementary file3 (TIF 144 KB) Supplementary Figure s1. Pie chart illustrating the distribution of different injuries in the study population, grouped in two main categories (clavicle fractures and fracture sequelae, acromioclavicular joint disorders) and classified according to the AO/OTA and the Rockwood classifications

Supplementary file4 (TIF 55 KB) Supplementary Figure s2. Pie chart illustrating the distribution of surgical procedures performed on the study population
