# Supplementary material for: The German version of the Nottingham Clavicle Score is a reliable and valid patient-reported outcome measure to evaluate patients with clavicle and acromioclavicular pathologies
Source: Knee Surg Sports Traumatol Arthrosc. 2022 Aug 29;31(5):1932–9. doi: 10.1007/s00167-022-07129-6 (PMC10090004; doi:10.1007/s00167-022-07129-6)
Supplement: Supplementary file 1 — Supplementary file1 (PDF 120 KB) [file 167_2022_7129_MOESM1_ESM.pdf]

Nottingham Clavicle Score – Deutsche Version

Die folgenden Fragen beziehen sich auf die Schmerzniveaus und Schwierigkeiten, die Sie in den letzten zwei Monaten im Bereich des Schlüsselbeins / der Schulter hatten.

1. Wie würden Sie die Schmerzen beschreiben, die Sie im Allgemeinen *an Ihrer Schulter / Ihrem Schlüsselbein* hatten?

◻	Keine	10
◻	Sehr mild	8
◻	Leicht	6
◻	Mäßig	4
◻	Stark	2

2. Wurden Sie nachts im Bett von Schmerzen *Ihrer Schulter / Ihres Schlüsselbeins* geplagt?

◻	Keine Nacht	10
◻	Nur 1 oder 2 Nächte	8
◻	Einige Nächte	6
◻	Die meisten Nächte	4
◻	Jede Nacht	2

3. In welchem Ausmaß haben *Schmerzen Ihrer Schulter / Ihres Schlüsselbeins* Ihre gewohnten Tätigkeiten (einschließlich Hausarbeit oder Autofahren) beeinträchtigt?

◻	Überhaupt nicht	10
◻	Ein bisschen	8
◻	Mäßig	6
◻	Außerordentlich	4
◻	Völlig	2

4. In welchem Ausmaß haben *Schmerzen Ihrer Schulter / Ihres Schlüsselbeins* Ihre sportlichen Aktivitäten oder Hobbys beeinträchtigt?

◻	Überhaupt nicht	10
◻	Ein bisschen/gelegentlich	8
◻	Manchmal	6
◻	Meistens	4
◻	Ständig	2

5. In welchem Ausmaß hat das *Problem mit Ihrer Schulter / Ihrem Schlüsselbein* Ihre Fähigkeit oder Bereitschaft zum Heben schwerer Gegenstände beeinträchtigt?

◻	Überhaupt nicht	10
◻	Gelegentlich	8
◻	Einige Tage	6
◻	An den meisten Tagen	4
◻	Jeden Tag	2

6. Ist Ihre Schulter / Ihr Schlüsselbein durch Überkopf-Aktivitäten leicht ermüdet oder hat sich geschwächt angefühlt?

◻	Überhaupt nicht	10
◻	Ein bisschen/gelegentlich	8
◻	Manchmal	6
◻	Meistens	4
◻	Ständig	2

7. Sind Sie mit dem Erscheinungsbild Ihres Schlüsselbeinbereichs zufrieden?

◻	Rundum zufrieden	10
◻	Sehr zufrieden	8
◻	Mäßig zufrieden	6
◻	Wenig zufrieden	4
◻	Überhaupt nicht zufrieden	2

8. Haben Sie Bewegungen oder ein Knacken im Schlüsselbeinbereich verspürt, welche Sie beunruhigen oder Ihnen Sorgen machen?

◻	Überhaupt nicht	10
◻	Ein bisschen/gelegentlich	8
◻	Manchmal	6
◻	Meistens	4
◻	Ständig	2

9. Verspüren Sie ein Kribbeln oder Taubheitsgefühl mit Ausstrahlung in den Nacken oder den Arm?

◻	Überhaupt nicht	10
◻	Ein bisschen/gelegentlich	8
◻	Manchmal	6
◻	Meistens	4
◻	Ständig	2

10. Haben Sie ein Ziehen oder ein Gefühl der Schwere Ihres Arms verspürt?

◻	Überhaupt nicht	10
◻	Ein bisschen/gelegentlich	8
◻	Manchmal	6
◻	Meistens	4
◻	Ständig	2

Übersetzung, kulturelle Anpassung in die deutsche Sprache und Validierung durch
Scheidt S, Zapatka J, Freytag RJ, Pohlentz MS, Paci M, Kabir K, Burger C, Cucchi D.
Universitätsklinikum Bonn, Klinik und Poliklinik für Orthopädie und Unfallchirurgie.

schulter@ukbonn.de
