# Supplementary material for: The German version of the Nottingham Clavicle Score is a reliable and valid patient-reported outcome measure to evaluate patients with clavicle and acromioclavicular pathologies
Source: Knee Surg Sports Traumatol Arthrosc. 2022 Aug 29;31(5):1932–9. doi: 10.1007/s00167-022-07129-6 (PMC10090004; doi:10.1007/s00167-022-07129-6)
Supplement: Supplementary file 2 — Supplementary file2 (DOCX 20 KB) [file 167_2022_7129_MOESM2_ESM.docx]

**Description of the scores used in the clinical trial**

**Nottingham Clavicle Score (NCS)**

The Nottingham Clavicle Score (NCS) is a 10-item PROM, specifically designed to measure outcomes after injuries and degenerative pathologies of the clavicle, acromioclavicular joint and sternoclavicular joint. The explored dimensions are pain (four items), strength/functional abilities (two items), cosmetic satisfaction (one item), mechanical symptoms such as movements or clicking (one item) and neurological symptoms in the upper limb such as tingling, numbness heaviness and dragging sensations (two items). The final score ranges from 20 to 100 points and can be graded as excellent (80–100), good (60–79), fair (40–59) or poor (<40).

**Imatani Score**

The Imatani Score was proposed in 1975 as a clinician reported outcome to evaluate acromioclavicular separations. It is composed of three items (pain, function, motion), with four possible response levels for pain (none, occasional, moderate, severe) and a five to ten points Likert scales for function and motion. The Imatani Score is calculated from the three items with maximum 40 points to pain, 30 points to function and 30 points to motion, ranging from zero points to one hundred. No validation of this score has ever been proposed.

**Disabilities of Arm, Shoulder and Hand (DASH)**

The Disabilities of Arm, Shoulder and Hand (DASH) was developed in 1996 for the evaluation of single or multiple musculoskeletal disorders affecting the upper limb; it is a patient-reported outcome measure which includes 30 items regarding symptoms, pain, physical function, and social function: 21 questions evaluate limitations and difficulties in performing specific activities, 6 questions assess specific symptoms and 3 questions measure social or occupational limitations. The DASH is considered one of the best tools for the comprehensive assessment of upper extremity, particularly in case of polyarticular conditions. As an anatomic-district score, it is not joint specific and should be combined with other outcome measurement instruments for evaluation of specific pathologies or injuries. A concept-retention version named QuickDASH was created by the developers and proved to be valid, reliable, responsive as the original version. A formula allows calculating the final score ranging from 0 (no disability) and 100 (the greatest possible disability).

**Oxford Shoulder Score (OSS)**

The Oxford Shoulder Score (OSS) was developed in 1996 specifically to assess shoulder pain and function in patients with shoulder operations other than stabilization. It is a multidimensional patient-reported outcome measure consisting in 12 items grouped in the following three domains: pain, function and social/psychological. Several translations of this short and easy-to-complete questionnaire are available, leading to an increasing diffusion in the orthopaedic literature. Each item offers 5 ordinal response options, scored from 0 to 4, with 4 representing the best. The 12 summed items produce an overall OSS from 0 to 48.

**Constant Murley Score (CMS)**

The Constant Murley Score (CMS) was developed in 1986 and published in 1987 to evaluate the general condition of healthy and pathological shoulders; it combines both patient- and clinician-reported scores and includes items regarding pain, activities of daily living, mobility, and strength. Relative weight of each domain is pain 15%, daily function and activity 20%, range of motion 40%, strength 25%. The CMS provides an overall clinical functional assessment, irrespective of diagnosis or radiographic abnormalities and is highly accepted throughout the orthopaedic community. Nevertheless, limited assessment of pain and limitations in everyday activities as well as a lack of standardisation in strength measurement are some limitations of this score. The CMS ranges from 0 to 100 points, representing worst and best shoulder function, respectively.

**EQ-5D-5L**

The EQ-5D is a family of instruments to describe and value health status and quality-of-life. The EQ-5D-5L measure is composed of five items (mobility, self-care, usual activity, pain/discomfort, and anxiety/depression). The EQ-5D index is calculated from the five dimensions, ranging from −0.594 (worst) to 1.0. Moreover, the EQ-5D includes a VAS for rating of overall health status from 0 (worst imaginable health) to 100 (best imaginable health). The EQ-5D-5L, used in this study, was introduced by the EuroQol Group in 2009 to improve the instrument’s sensitivity, improving sensitivity to change and reducing ceiling effects, as compared to the previous EQ-5D-3L version.
