# Supplementary figures and images for: The German version of the Nottingham Clavicle Score is a reliable and valid patient-reported outcome measure to evaluate patients with clavicle and acromioclavicular pathologies
Source: Knee Surg Sports Traumatol Arthrosc. 2022 Aug 29;31(5):1932–9. doi: 10.1007/s00167-022-07129-6 (PMC10090004; doi:10.1007/s00167-022-07129-6)

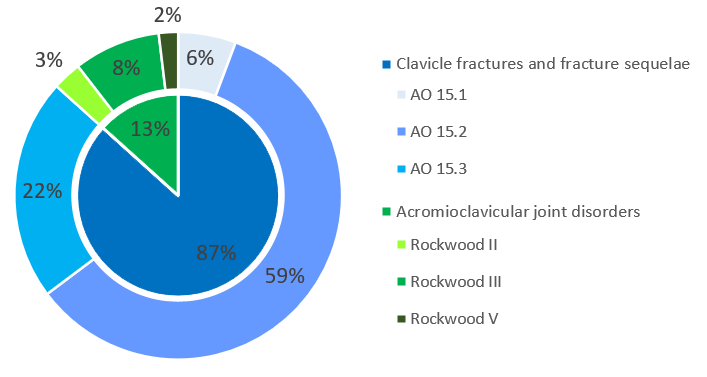

Supplement: Supplementary file 3 — Supplementary file3 (TIF 144 KB) Supplementary Figure s1. Pie chart illustrating the distribution of different injuries in the study population, grouped in two main categories (clavicle fractures and fracture sequelae, acromioclavicular joint disorders) and classified according to the AO/OTA and the Rockwood classifications [file 167_2022_7129_MOESM3_ESM.tif]

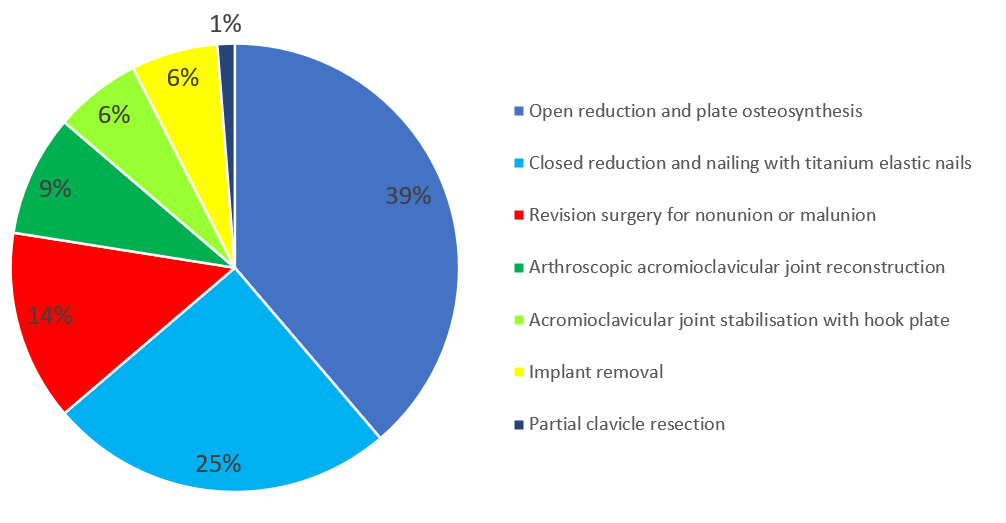

Supplement: Supplementary file 4 — Supplementary file4 (TIF 55 KB) Supplementary Figure s2. Pie chart illustrating the distribution of surgical procedures performed on the study population [file 167_2022_7129_MOESM4_ESM.tif]
